# A Canadian Study toward Changing Local Practice in the Diagnosis of Pediatric Celiac Disease

**DOI:** 10.1155/2016/6234160

**Published:** 2016-04-26

**Authors:** Seema Rajani, Hien Q. Huynh, Leanne Shirton, Cheryl Kluthe, Donald Spady, Connie Prosser, Jon Meddings, Gwen R. Rempel, Rabindranath Persad, Justine M. Turner

**Affiliations:** ^1^Department of Pediatrics, University of Alberta, Edmonton, AB, Canada T6G 1C9; ^2^Multidisciplinary Pediatric Celiac Clinic, Stollery Children's Hospital, Edmonton, AB, Canada T6G 2B7; ^3^Department of Laboratory Medicine and Pathology, Department of Medicine, University of Alberta, AB, Canada T6G 2R3; ^4^Cumming School of Medicine, University of Calgary, Calgary, AB, Canada T2N 4Z6; ^5^Faculty of Health Disciplines, Athabasca University, Athabasca, AB, Canada T9S 3A3

## Abstract

*Background*. The European Society for Pediatric Gastroenterology, Hepatology and Nutrition endorses serological diagnosis (SD) for pediatric celiac disease (CD). The objective of this study was to pilot SD and to prospectively evaluate gastrointestinal permeability and mucosal inflammation at diagnosis and after one year on the gluten-free diet (GFD). We hypothesized that SD would be associated with similar short term outcomes as ED.* Method*. Children, 3–17 years of age, referred for possible CD were eligible for SD given aTTG level ≥200 U/mL, confirmed by repeat aTTG and HLA haplotypes. Gastrointestinal permeability, assessed using sugar probes, and inflammation, assessed using fecal calprotectin (FC), at baseline and after one year on a GFD were compared to patients who had ED.* Results*. Enrolled SD (*n* = 40) and ED (*n* = 48) patients had similar demographics. ED and SD groups were not different in baseline lactulose: mannitol ratio (L : M) (0.049 versus 0.034; *p* = 0.07), fractional excretion of sucrose (%FES; 0.086 versus 0.092; *p* = 0.44), or fecal calprotectin (FC; 89.6 versus 51.4; *p* = 0.05). At follow-up, urine permeability improved and was similar between groups, L : M (0.022 versus 0.025; *p* = 0.55) and %FES (0.040 versus 0.047; *p* = 0.87) (*p* > 0.05). FC improved but remained higher in the SD group (37.1 versus 15.9; *p* = 0.04).* Conclusion*. Patients on the GFD showed improved intestinal permeability and mucosal inflammation regardless of diagnostic strategy. This prospective study supports that children diagnosed by SD have resolving mucosal disease early after commencing a GFD.

## 1. Introduction

Celiac disease (CD) is a common autoimmune gastrointestinal disorder, with an estimated prevalence of 1 : 133 in North America [1, 2]. It is triggered by ingestion of gluten and causes increased small intestine permeability and enteropathy. CD is known to occur through a genetic predisposition, the HLA associated genes being necessary but insufficient to cause CD [3]. In North America, guidelines from the North American Society of Pediatric Gastroenterology, Hepatology and Nutrition (NASPGHAN) recommend histological confirmation through biopsy of the small intestine as the gold standard for CD diagnosis [4]. However, high level titers of antitissue transglutaminase antibodies (aTTG) are well recognized to be associated with positive histology [5–12]. Thus, the necessity of biopsy has been questioned for pediatric patients. Furthermore, an increase in CD prevalence and recognition of atypical presentations raises the need for affordable and rapid diagnosis. This is becoming problematic in many institutions because of shrinking endoscopy time and resources and hence increasing waitlists. In 2012, the European Society of Pediatric Gastroenterology, Hepatology and Nutrition (ESPGHAN) updated their diagnostic guidelines to include serological diagnosis (SD) [5].

The purpose of this study was to pilot SD in our local clinic. We applied stringent modified ESPGHAN criteria. We hypothesized that SD would lead to similar clinical outcomes in patients after one year on a gluten-free diet (GFD), compared to biopsy and endoscopic diagnosis (ED). Our study objective was to measure intestinal permeability and inflammation at baseline and after one year on the GFD in order to demonstrate improvement in mucosal disease, occurring independently of the diagnostic approach. This would be the first publication of a prospective study of patients whose diagnosis was based on SD. This study was not designed to prove the diagnostic accuracy (hence, safety) of SD as this has been published already and has led to the current European pediatric guidelines [5].

## 2. Methods

**Figure 1:**
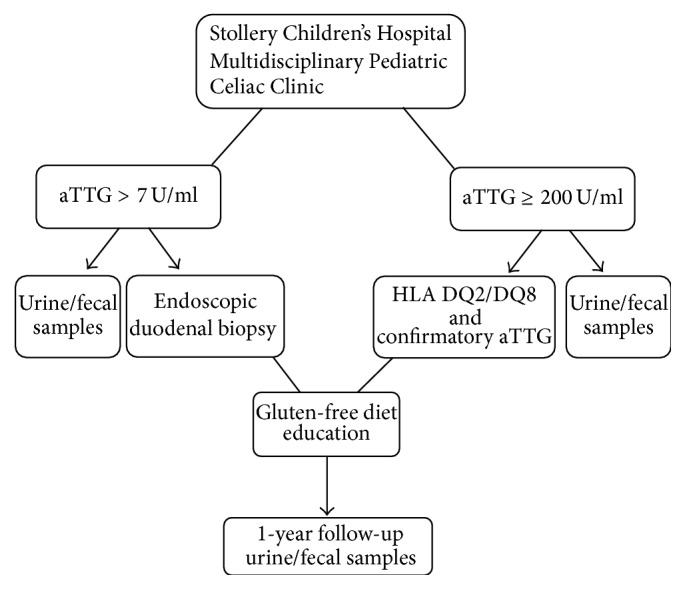
Study design.

Consecutive patients, 3–17 years old, were recruited from the multidisciplinary celiac disease clinic at Stollery Children's Hospital, after referral for an elevated aTTG (>7.0 U/mL) (Figure 1). Patients were excluded if they had diabetes or language barriers or were off dietary gluten. Ethical approval was granted by the University of Alberta Research Ethics Board and all subjects provided informed consent.

### 2.1. Serological Diagnosis

Patients were eligible to consent to SD if they met our modification of the ESPGHAN criteria, justified as follows:

1. An aTTG level ≥ 200 U/mL was used as the cut-off for eligibility. This is based on our own research using the same local laboratory method (using EliA Celikey IgA tTG) [9]. ESPGHAN criteria use aTTG 10x the upper limit of normal (ULN), which would be ≥70 U/mL based on our laboratory normal value [5]. However, in our experience this would have led to the misdiagnosis of 2 patients (2/115) [9]. Given that this was a pilot study, it was essential in our opinion to use the most conservative cut-off and we used 28x ULN based on the prior demonstrated specificity [9].
2. ESPGHAN criteria call for a confirmatory test, recommending antiendomysial antibody (EMA), from a blood sample drawn at an occasion separate from the initial aTTG test to avoid false positive results, owing to mislabeling or other technical errors [5]. In our laboratory in Edmonton we do not currently have access to EMA, as a quality assurance process in the laboratory had identified the false positive rate of aTTG to be 0.3% [13]. Therefore, a second confirmatory aTTG test was undertaken from a separate blood draw, done at the time of HLA testing. On this occasion we accepted an aTTG > 10x ULN, consistent with ESPGHAN criteria. This was because in our experience it is common for families to reduce gluten intake following the first aTTG result.
3. Asymptomatic patients were not excluded, as was the case in our published study [9], and the inclusion of asymptomatic patients is supported by recently published data [14].

SD required confirmation of at-risk HLA haplotypes for CD (DQ2, DQ8), performed using reverse Sequence Specific Oligonucleotide (rSSO) and fluorescence measured by Luminex software. The Luminex analyzer is a flow cytometer with the ability to test multiple antigens in one well. The aTTG assays were done through the laboratory at the University of Alberta Hospital, the primary hub for Northern and Central Alberta, using ELiA Celikey IgA (Phadia AB, Sweden), with a five-point calibration curve.

### 2.2. Endoscopic Diagnosis

Consecutive patients who had aTTG < 200 U/mL or did not consent to SD and so were having diagnostic upper endoscopy and biopsy were the comparison (ED) group. Routinely, 6 samples at the distal duodenum and 2 samples at the duodenal bulb were obtained. Two pediatric pathologists designated Marsh scores. Diagnosis of CD was based on clinicopathological correlation that included Marsh 2/3 scores.

### 2.3. Gastrointestinal Permeability

Noninvasive measurements using orally administered sugar probes (lactulose, mannitol, and sucrose) were used to determine gastrointestinal permeability at diagnosis and after one year on the GFD [15–18]. Urine samples were also collected from healthy individuals with no gastrointestinal symptoms or family history of CD [19]. Neither patients nor controls were taking any medications known to alter permeability. Sugar probes were administered in Kool-Aid (Kraft, Northfield, IL) and dosed according to weight. Overnight urine was collected, total volume was recorded, and aliquots of urine were stored at −80°C. Analysis of lactulose-to-mannitol ratio (L : M) and fractional excretion of sucrose (% FES) was done by high-performance liquid chromatography (HPLC), adjusted for urine weight and volume [20].

### 2.4. Mucosal Inflammation

Fecal calprotectin (FC) was measured at diagnosis and after one year on the GFD. A first morning stool was collected and kept frozen at −80°C until it was analyzed using Immundiagnostik AG enzyme-linked immunosorbent assay (ELISA) kit (Bensheim, Germany). The laboratory cut-off value for FC in children aged 4–17, which is below 50 *μ*g/g, was used as the control value [21].

### 2.5. Adherence

Adherence to the gluten-free diet was assessed by a dietitian focused interview at the annual follow-up appointment [22]. Adherence was supported by symptom improvement (reported to a physician using a standardized questionnaire) and also by improvement of aTTG levels.

### 2.6. Statistical Analysis

SPSS 22 was used for data analysis. Independent sample *t*-tests were used to determine differences between diagnostic groups for normally distributed demographic variables, symptom improvement, adherence, aTTG decline from baseline to follow-up, and percentage of aTTG normalized. Given skewed distribution, the noninvasive measurements (L : M, %FES, and FC) were compared using nonparametric analysis (Mann-Whitney or Wilcoxon Signed Rank Test). Alpha was set at 0.05.

## 3. Results

From January 2013 to June 2014, 118/170 eligible patients were recruited, 53/71 eligible for SD and 65/99 eligible for ED. Subsequently, 30 patients were excluded or lost to follow-up (13 SD, 17 ED). Exclusions were due to negative or Marsh 1 biopsies (9, all aTTG < 200 U/mL), negative or missing genetic result (2), or patients that were not contactable after consent at the initial clinic visit (19). A total of 88 patients were enrolled in the study, 40 with SD and 48 with ED.

### 3.1. Baseline Demographics

Patient demographics are compared in Table 1. There was no difference in age, gender, height, or weight between diagnostic groups. One SD and one ED patient were asymptomatic, both screened due to family history. Overall, 48% of SD patients and 43% of ED patients reported a family history of CD. Gastrointestinal symptoms predominated (88% SD and 85% ED). Other miscellaneous reported symptoms included headache, irritability, mood disturbance, foggy mind, paresthesias, or tremors. HLA haplotype distribution for the SD group was 70% DQ2/DQX, 10% DQ8/DQX, 15% homozygous DQ2, and 5% homozygous DQ8. Marsh score distribution for the ED group was 81% Marsh 3 and 19% Marsh 2. As expected, baseline aTTG was higher in SD patients (595 versus 42 U/mL; *p* < 0.001).

### 3.2. One-Year Follow-Up on a Gluten-Free Diet

Thirty-two SD and 43 ED patients were seen at 12-month follow-up and again there were no significant differences other than aTTG (Table 1). At follow-up, aTTG was higher for SD than ED (9 versus 4 U/mL; *p* = 0.005), although it declined at a greater rate in SD compared to ED (98% versus 91%; *p* < 0.001). Fewer SD patients had normal aTTG at follow-up (40% versus 72% < 7.0 U/mL; *p* = 0.01). Symptom improvement and adherence to the GFD did not differ between the groups (Table 1).

### 3.3. Permeability

At baseline, CD patients had increased L : M and % FES compared to controls, which is not different between the groups. There were no follow-up differences between SD, ED, and control (Table 2).

### 3.4. Fecal Calprotectin

At baseline, the SD had higher FC compared to ED and the expected laboratory cut-off. At follow-up, all patients had a decline in FC; in the SD group, FC remained higher than for ED but was not different from control (Table 3).

## 4. Discussion

This study is the first prospective evaluation of pediatric patients diagnosed by SD using noninvasive monitoring of permeability and inflammation and comparing to patients diagnosed by ED. Using such noninvasive biomarkers of mucosal disease, we found that SD patients had abnormalities at diagnosis, consistent with CD, and at follow-up they improved. Hence, in our local clinic, our approach to SD did not appear to disadvantage short term outcomes for these children, who also had similar dietary adherence and symptom improvement as ED patients during follow-up.

Although ESPGHAN has endorsed a SD approach, such an approach remains controversial in North America. Yet, according to laboratory data in our health region, one third of children with a positive CD serological screen are not referred for a confirmatory biopsy [9]. In many cases the children had aTTG levels below the recommended threshold by ESPGHAN [5]. We concluded that in our local community either physicians, patients, or both do not always want a biopsy to confirm CD. These children do not receive appropriate diagnostic confirmation and are also missing out on the support and education our clinic provides [4, 23, 24]. Hence, we undertook to pilot a SD approach in our clinic. We aimed to show that we could apply SD criteria and with careful follow-up would see improvements expected for a child with CD on a GFD. While this may seem quite obvious, it has in fact not been published previously.

At enrollment and follow-up, the main differences between diagnostic groups were aTTG levels. More ED patients had normalized aTTG at 12-month follow-up. This can be explained by the higher aTTG levels in SD patients at diagnosis requiring more time to decrease to normal. Hogen Esch et al. have reported 80% of CD patients to be serologically negative for aTTG after 2 years on the GFD [25]. However, the rate of decline of aTTG was higher in the SD group. Despite the small residual aTTG difference, such rate of decline, the improvement in symptoms, and the improvement in permeability and FC results, all suggest a positive effect of the GFD in the SD group.

The L : M ratio is considered a measure of permeability throughout the small bowel, while % FES is more representative of upper small intestine permeability [26, 27]. As expected, both were elevated at baseline and improved in both diagnostic groups on the GFD [28–30]. FC was more elevated in SD at baseline and also at follow-up, suggesting potentially more mucosal inflammation in the SD group. Previous studies have shown that patients with increased levels of aTTG have a higher probability of increased damage represented by Marsh scores [10, 11, 31]. A higher FC level in patients with increased aTTG levels has also been shown by Ertekin et al. [32].

A limitation of this study was the poor return rate of stool and urine samples, especially for the ED group, and this introduces a potential bias. It was also disappointing considering the wide range/variability of these results. However, it was reassuring that all individuals who provided baseline and follow-up samples showed improvement (especially for the SD patients, who were more likely to return samples). Unfortunately, these tests appear to have poor acceptability in the clinical setting and this will limit their utility to follow SD patient's long term. Criticisms of this study will include that we modified the ESPGHAN approach to SD. The ESPGHAN guidelines recognize interlaboratory variability with aTTG testing and so proposed aTTG 10x ULN for SD. We would argue that understanding how our local aTTG assay compared to biopsy findings in our population was in fact a strength of our study, enabling determination of the best or “safest” local cut-off for SD. We used a cut-off of 28x ULN for SD as we knew this to have very high specificity, avoiding false positive diagnoses [9]. We recognize that with increased specificity there will be reduced sensitivity, but below the cut-off we biopsy all patients with aTTG > 7 U/mL, avoiding the risk of false negatives.

We were not able to use a confirmatory EMA test. The SD approach, as endorsed by ESPGHAN, is in fact based on aTTG and not on EMA levels. We took the pragmatic approach of using a second aTTG test to exclude the possibility of rare laboratory handling errors. Other studies have shown that EMA is consistently positive with aTTG levels ≥ 100 U/mL [8, 9, 33]. Brusca et al. also showed that all aTTG and EMA serological combinations in their study were equivalent to aTTG alone [34]. Other studies have compared both tests and determined that aTTG is as reliable as EMA, if not better [35, 36].

Finally, another modification we supported was the inclusion of asymptomatic patients. Asymptomatic patients have increased risk of CD related morbidity, like osteoporosis, and also face the same burden of a GFD [37–39]. They often report relief of extraintestinal manifestations, such as fatigue and irritability, on the diet [40]. We did not believe it reasonable to exclude them given a very high aTTG. However, in the end we had very few asymptomatic patients included and so cannot make conclusions about this group of patients. Recently, Trovato et al. [14] suggest that it is reasonable to include asymptomatic patients for SD as they found no histological differences compared to symptomatic patients when applying ESPGHAN criteria. Again, more studies are required confirming their findings. Patients with diabetes were not offered SD due to well-known fluctuating aTTG levels and the possibility of normalization of aTTG levels even on gluten [41].

The major limitations of this study are the small sample size and limited duration of follow-up. Also, this study did not randomize patients to SD when eligible (aTTG > 200 U/mL and HLA haplotypes) and so there is a risk of self-selection bias. Despite this, we showed no adverse effect of SD in children diagnosed at a tertiary referral clinic in North America. ESPGHAN criteria are based on studies comparing aTTG to biopsy using retrospective study designs. We believe it is essential, before considering a major change in clinical practice in North America, that prospective studies in SD diagnosed patients also be undertaken. Our approach was customized for our own laboratory and local community and thus cannot be readily applied to other centers [9]. However, it is our opinion that a “one size fits all” approach may not be best. Such an approach does not eliminate potential impact of variability in aTTG diagnostic kits across laboratories. It is the current approach of our clinic to offer potential SD to patients with an aTTG ≥ 200 U/mL. Using the information of this study and our prior research, we inform parents/caregivers and children of our experience and of the current pediatric guidelines (both ESPGHAN and NASPGHAN) and allow them freedom of choice.

In North America, the SD approach is criticized because of the potential for misdiagnosis or the possibility that the diagnostic approach might be less firmly “believed” than ED, leading to poor dietary adherence. In our study, we have shown an improvement in symptoms, mucosal permeability, and inflammation that would argue against misdiagnosis, and we did not see any negative impact on adherence. We hope that in implementing, testing, and sharing our positive experience of pediatric SD local patients not desiring a biopsy will still be referred to our tertiary clinic. We also hope that current NASPGHAN guidelines for CD diagnosis, dated to 2005, will undergo revision and that this study will add data to support SD [4]. Finally, we hope this study will prompt more prospective studies to be undertaken.

## Figures and Tables

**Table 1:** Baseline and follow-up comparisons between diagnostic groups.

		Serological diagnosis (*n* = 40)	Biopsy diagnosis (*n* = 48)	*p* value
Baseline	Age (years)^1^	8.6 (3.5)	9.2 (3.5)	0.38
	Gender (M : F)	16 : 24	18 : 30	0.81
	Height *z*-score^1^	−0.05 (1.04)	0.05 (0.99)	0.66
	Weight *z*-score^1^	−0.03 (1.00)	0.04 (1.03)	0.73
	aTTG (U/mL)^2^	595 (200–4100)	42 (7.8–2500)	<0.001
	GI symptoms	87.5%	85%	1.0
	Anemia/fatigue	65%	54%	0.38
	Symptoms			
	Family history	47.5%	43%	0.67

		(*n* = 32)	(*n* = 41)	
Follow-up	Diagnosis to follow-up (months)^1^	11.0 (3.8)	11.7 (12.16)	0.42
	Age (years)^1^	9.5 (3.5) (*n* = 32)	10.5 (3.7)	0.26
	Gender (M : F)	14 : 18	16 : 25	0.97
	Height *z*-score^1^	−0.11 (1.04)	0.10 (1.00)	0.42
	Weight *z*-score^1^	−0.11 (0.93)	0.10 (1.07)	0.37
	GFD adherence	94%	93%	0.43
	Symptom improvement	93%	85%	0.52
	aTTG (U/mL)^2^	9.4 (1–98)	4.0 (1–420)	0.005
	% aTTG decline^2^	98.5 (90.7–99.9)	91.4 (308–99.8)	<0.001
	aTTG < 7 U/mL%	40%	72%	0.013

^1^Mean (standard deviation); ^2^median (range).

**Table 2:** Permeability results.

		All celiac patients	Serological diagnosis (*n* = 38)	Biopsy diagnosis (*n* = 27)	Control (*n* = 26)
Baseline	L : M	0.043^*∗*^ (0.011–0.29)	0.049^*∗*^ (0.022–0.292)	0.034^*∗*^ (0.011–0.155)	0.022 (0.010–0.072)
	% FES	0.087^*∗*^ (0.003–0.448)	0.086^*∗*^ (0.011–0.448)	0.092^*∗*^ (0.003–0.27)	0.045 (0.010–0.530)

			(*n* = 30)	(*n* = 17)	(*n* = 26)
Follow-up	L : M	0.024 (0.011–0.317)	0.022 (0.012–0.317)	0.025 (0.011–0.042)	0.022 (0.010–0.072)
	% FES	0.044 (0.00–0.878)	0.040 (0.00–0.259)	0.047 (0.00–0.878)	0.045 (0.010–0.530)

Data is expressed as median and range.

% FES, percentage fractional excretion of sucrose; L : M, lactulose-to-mannitol ratio.

^*∗*^Significantly different from control (*p* < 0.05, Mann-Whitney test or Wilcoxon Signed Rank Test).

**Table 3:** Fecal calprotectin results.

	All celiac patients	Serological diagnosis (*n* = 38)	Biopsy diagnosis (*n* = 27)	Laboratory normal cut-off
Baseline^§^	67.5^*∗*^ (4.9–3068)	89.6^*∗*^ (6.0–3068)	51.4 (4.9–1755)	<50

		(*n* = 30)	(*n* = 16)	
Follow-up^§^	33 (1.11–736.5)	37.1 (5.7–319.5)	15.9 (1.11–736.5)	<50

Data is expressed as median and range.

^*∗*^Significantly different from normal laboratory cut-off (<50) (*p* < 0.05, Mann-Whitney test or Wilcoxon Signed Rank Test).

^§^Significantly different between diagnostic groups (*p* < 0.05, Mann-Whitney test).
